# Supplementary material for: Time-Course Responses of Apple Leaf Endophytes to the Infection of Gymnosporangium yamadae
Source: J Fungi (Basel). 2024 Feb 3;10(2):128. doi: 10.3390/jof10020128 (PMC10890309; doi:10.3390/jof10020128)
Supplement: Supplementary file 1 [file jof-10-00128-s001.zip › Supplementary figures and tables.pdf]

Table S1. Annotation of all sequences in all samples at various taxonomic levels and ASVs.

Sample	Kingdom	Phylum	Class	Order	Family	Genus	Species	OTU
bacteria	1	37	98	160	261	551	376	10152
fungi	1	4	12	26	42	43	29	4123

Table S2. Low abundance taxa at the bacterial phylum level in each sampling point (ranked 20th and beyond in terms of abundance among all taxa).

Phylum	diseased_6h	diseased_24h	diseased_3d	diseased_5d	diseased_11d	diseased_14d	diseased_20d	diseased_70d	diseased_90d
Chloroflexi	0.001847328	7.92E-05	0	8.86E-05	0.000956111	0.022474754	0.010706722	0.00030698	0.000903586
Bdellovibrionota	0.001423408	8.97E-05	0.00021824	0.000408523	0.000198707	0.00150949	0.002047527	0.00122637	0.004152385
Myxococcota	0.000875509	0.000176453	0.00050126	0.000183456	0.000187155	0.0046654	0.003299577	0.002065139	0.000722768
Desulfobacterota	0.000816367	0	0.00014298	0.000294641	0.000125154	0.007999244	0.004134508	0.000659916	0
Deinococcota	0.000941672	5.48E-05	3.01E-05	0.000412012	0.000120768	0.000206709	0.000569523	0.002712669	0.005517733
Armatimonadota	0.000132639	2.96E-05	0	0.000262727	9.02E-05	0.00026734	0.000119772	0.00016769	0.004441327
Verrucomicrobiota	0.000647924	1.76E-05	0.00013544	0.000172337	0.000233673	0.004668615	0.003854182	0.000302018	0
Gemmatimonadota	0.000162227	0	0.00011461	1.36E-05	0.000179544	0.006437728	0.002247704	0.000144357	4.56E-05
Abditibacteriota	0	0	0	0.000265008	0	0.000206214	0.000248099	0.000761593	0.007706625
Nitrospirota	0.000185283	0.000121142	0	0	0.000167395	0.003398942	0.003336911	0.000461437	0.000106341
Planctomycetota	0.000173491	0.000149694	0	0	0.000184721	0.002640515	0.001149031	0.000598049	0
Dependentiae	7.30E-05	0	0.00027841	0	0	0.001311843	0	0	4.19E-05
Fusobacteriota	0.000212044	0	4.31E-05	0	0	0.000672988	0.000136882	0.000134259	0
Elusimicrobiota	7.42E-05	0	6.16E-05	0.000216811	0	0.000412429	0.001121604	0.00052613	7.52E-05
NB1_j	2.10E-05	1.48E-05	0.00014981	0	0	0.000472356	0.000767226	9.17E-05	5.24E-05
Other	0.002059552	0.000670499	0.00028391	0.000177301	0.000345381	0.007332592	0.006029252	0.000424055	4.66E-05

Phylum	healthy_6h	healthy_24h	healthy_3d	healthy_5d	healthy_11d	healthy_14d	healthy_20d	healthy_70d	healthy_90d
Chloroflexi	0	0.001227172	0.00232728	0.001191956	0.000104552	0.00053184	0.00156522	0.012518753	0.000344525
Bdellovibrionota	0.002797073	0.00279135	0.00125571	0.001189554	0.002305952	0.001126752	0.000545918	0.002363565	0.005419279
Myxococcota	0.000974259	0.000864432	0.00294246	0.000745036	0.002203031	0.000886609	0.00103496	0.003291598	0.003931118
Desulfobacterota	0.000317788	0.000452309	0.00055393	0.00012848	0.000510263	0.000785498	0.00040599	0.004112245	7.97E-05
Deinococcota	0.001664372	0.000269592	0.00023312	0.000415346	0.000133156	5.53E-05	0.002441331	0.000353581	0.002527446

Armatimonadota	0.00218155	0.000142794	6.17E-05	0.007105436	0	0.000210821	0	0.000680093	0.001367487
Verrucomicrobiota	0.000806395	0.000178992	0	0	0.000628558	0.000809187	0.001455958	0.001463867	0.000185908
Gemmatimonadota	0.000101128	0.000383618	0.00068134	0.000143317	1.12E-05	0.000816387	0	0.002358205	6.89E-05
Abditibacteriota	0	0.000119947	0	0.001673012	0	0.001122742	0	0	0.001186271
Nitrospirota	0.001647446	0.000271839	0	0	0.00034662	0	0.001026915	0.000120034	0
Planctomycetota	0.000144469	0.000271985	0.0013948	0	0	0	0.000150908	0.000271214	0.00050238
Dependentiae	0.000330994	0.000295464	0.00070998	0	0.001335072	0	0.000669292	0	0
Fusobacteriota	0.00046291	7.89E-05	0.00014903	0.000407285	0.000147635	0.001043643	0.000283162	0	0
Elusimicrobiota	6.02E-05	0.000176965	0.00060542	0	0	0	0.000193065	0.000474978	0
NB1_j	0	0.000256463	0	0	0.000464969	0	0.000271635	0.000948945	0
Other	0.001149294	0.001301437	0.00024068	0.000634199	0.000636018	0	0.00053894	0.002725284	8.86E-05

Table S3. Low abundance taxa at the bacterial class level in each sampling point (ranked 20th and beyond in terms of abundance among all taxa).

Class	diseased_6h	diseased_24h	diseased_3d	diseased_5d	diseased_11d	diseased_14d	diseased_20d	diseased_70d	diseased_90d
Bacteroidia	0.01864567	0.01025847	0.00608732	0.00795762	0.0208291	0.06405695	0.06573949	0.01690357	0.02040729
Thermoleophilia	0.00224116	0.00033422	0.00307307	0.00178269	0.00145125	0.03367159	0.01469781	0.0033328	0.00244854
Acidobacteriae	0.00090534	0.00054567	0.00110664	0.00046698	0.00049876	0.01115474	0.00467069	0.0038426	0.00051146
Acidimicrobiia	0.00012819	6.23E-05	0.00083163	0.00013715	0.00010024	0.01028707	0.00454476	0.00045809	0.00017053
Bdellovibrionia	0.00096366	6.23E-05	8.28E-05	0.00021347	7.02E-05	0.0008661	0.00111362	0.00119478	0.00353312
Polyangia	0.00076327	0.00015884	0.00026902	0.00015566	0.00013704	0.0033411	0.00259252	0.00095718	0.00027462
AD3	0	0	0	0	5.01E-05	0.00900325	0.00545299	0	0
Deinococci	0.00094167	5.48E-05	3.01E-05	0.00041201	0.00012077	0.00020671	0.00056952	0.00271267	0.00551773
Fimbriimonadia	0.00013264	2.96E-05	0	0.00026273	9.02E-05	0.00026734	0.00011977	0.00016769	0.00410711
MB_A2_108	0	2.96E-05	0.00016214	0	0.00027279	0.00351208	0.00561934	0	0
Limnochordia	0	0	0	0	0.00037088	0.00563505	0.00759239	0	0
TK10	0	0	0	0	8.02E-05	0.00415309	0.00216775	0	0.00010485
Gemmatimonadetes	0.00013669	0	6.38E-05	1.36E-05	0.00014947	0.00619244	0.00200108	0.00014436	0
Abditibacteria	0	0	0	0.00026501	0	0.00020621	0.0002481	0.00076159	0.00770663
unclassified	0.00075957	0.00018453	0	0	0	0.0008865	0.00202546	0.00043994	0.00353765
Other	0.00963474	0.00184514	0.00231822	0.00170905	0.00277583	0.05118783	0.03235216	0.00628924	0.00291553

Class	healthy_6h	healthy_24h	healthy_3d	healthy_5d	healthy_11d	healthy_14d	healthy_20d	healthy_70d	healthy_90d
Bacteroidia	0.03533521	0.03826801	0.03673052	0.01390706	0.02181452	0.03562631	0.0588622	0.04827124	0.05024898
Thermoleophilia	0.00467204	0.00449386	0.00374468	0.00808934	0.00118318	0.00401224	0.00371092	0.01681338	0.00042995
Acidobacteriae	0.00346688	0.00118795	0.00532147	0.00074287	0.00141542	0.00232793	0.00324489	0.00909536	0.00122928
Acidimicrobiia	0.00090992	0.00124456	0.00019806	0.00059541	0.00102683	0.00152927	0.00039485	0.00682691	0.00059933
Bdellovibrionia	0.00237751	0.00251063	0.00085226	0.00100513	0.00131523	0.00086235	0.00054592	0.00192752	0.00435445
Polyangia	0.00046272	0.00065101	0.00241622	0.0005975	0.00220303	0.00073967	0.00077226	0.00277049	0.00153165
AD3	0	0	0	0	0	0	0	0.0053182	0
Deinococci	0.00166437	0.00026959	0.00023312	0.00041535	0.00013316	5.53E-05	0.00244133	0.00035358	0.00252745

Fimbriimonadia	0.00218155	0.00014279	6.17E-05	0.00710544	0	0.00021082	0	0.00068009	0.00136749
MB_A2_108	0.0003395	0.00146335	0	0.00035049	0	0.0016743	0	0.00200892	0
Limnochordia	0	0	0	0	0	0	0	0.00070079	0
TK10	0	0.00070291	0.00218111	0.0005188	0	0.00029207	0	0.00244491	6.89E-05
Gemmatimonadetes	4.33E-05	0.00026871	0.00068134	0.00014332	0	0.0005721	0	0.00218892	6.89E-05
Abditibacteria	0	0.00011995	0	0.00167301	0	0.00112274	0	0	0.00118627
unclassified	0.00081625	0.00010023	2.09E-05	0	0.000348	0.0002677	0.00040599	0.00098206	0
Other	0.00694707	0.00582844	0.00516027	0.00272639	0.00539086	0.00458926	0.00694975	0.02034713	0.00703531

Table S4. Low abundance taxa at the fungal phylum level in each sampling point (ranked 20th and beyond in terms of abundance among all taxa).

name	diseased_6h	diseased_24h	diseased_3d	diseased_5d	diseased_11d	diseased_14d	diseased_20d	diseased_70d	diseased_90d
Ascomycota	0.764599339	0.484791526	0.619890296	0.514730729	0.763981195	0.825546438	0.636687252	0.56601028	0.681654128
Basidiomycota	0.006511188	0.014042673	0.009844644	0.011366461	0.013905674	0.00480353	0.011969271	0.002200219	0.003130325
Mortierellomycota	0	0	0	0	0	0	0	0.000392619	0
Rozellomycota	0.000327577	0	0	0.000938764	0	0	0	0.001359802	0
unclassified	0.307562765	0.501165801	0.37026506	0.472964046	0.222113131	0.169650032	0.351343477	0.43003708	0.532720909

name	healthy_6h	healthy_24h	healthy_3d	healthy_5d	healthy_11d	healthy_14d	healthy_20d	healthy_70d	healthy_90d
Ascomycota	0.889392732	0.796216466	0.78272912	0.742425317	0.861845954	0.686115383	0.489002504	0.417531354	0.512898576
Basidiomycota	0.00528348	0.026763774	0.017504094	0.008165167	0.004040991	0.007038348	0.022849032	0.01352015	0.007508982
Mortierellomycota	0	0.000270022	0	0	0	0	0	0	0
Rozellomycota	0	0	0	0	0	0.000967697	0	0	0
unclassified	0.105323788	0.176749738	0.199766786	0.334933347	0.134113055	0.305878573	0.488148464	0.680375735	0.683134952

Table S5. Low abundance taxa at the fungal class level in each sampling point (ranked 20th and beyond in terms of abundance among all taxa).

name	diseased_6h	diseased_24h	diseased_3d	diseased_5d	diseased_11d	diseased_14d	diseased_20d	diseased_70d	diseased_90d
Dothideomycetes	0.002848321	0.01943549	0.11593811	0.04400194	0.026355838	0.006158407	0.019237643	0.496687072	0.449142732
Eurotiomycetes	0.058787657	0.04168089	0.12389434	0.02709832	0.162208072	0.040749906	0.151423254	0.031613569	0.000705419
Leotiomycetes	0	0.00359988	0.00011819	0.00131204	0.000199866	0	0.000347737	0	0
Pezizomycetes	0	1.7576E-05	0.00239046	0	0.001977341	0	0.001421916	0.001175965	0
Saccharomycetes	0.001169919	0	0	0	0.001579403	2.88157E-05	0.004597685	0	4.59464E-05
Sordariomycetes	0.003608576	0.00134785	0.02656974	0.03311592	0.003883664	0.004763893	0.02039573	0.009777447	0.010401599
Agaricomycetes	0.00647375	0.00857248	0.00441813	0.00436835	0.01361513	0.001598531	0.010620004	0.002200219	0.00173186
Cystobasidiomycetes	0	0.0022497	0.00336951	0.00024873	0	0	0	0	9.30238E-05
Malasseziomycetes	3.74374E-05	0.00016231	0.00052899	5.7045E-05	0.000290544	0.001620133	0.000321509	0	0
Tremellomycetes	0	0.00305818	0.0006838	0	0	0	0.001027757	0	2.32559E-05
Mortierellomycetes	0	0	0	0	0	0	0	0.000392619	0
Rozellomycotina	0.000327577	0	0	0.00093876	0	0	0	0.001359802	0
unclassified	0.926746762	0.91987565	0.72208873	0.8888589	0.789890143	0.945080314	0.790606765	0.456793308	0.537856163

name	healthy_6h	healthy_24h	healthy_3d	healthy_5d	healthy_11d	healthy_14d	healthy_20d	healthy_70d	healthy_90d
Dothideomycetes	0.003472178	0.02595918	0.04087869	0.04438286	0.029151206	0.008331235	0.003964312	0.194570851	0.483528556
Eurotiomycetes	0.255794104	0.241981	0.49383528	0.26833957	0.032098955	0.062967283	0.092859585	0.056968022	0.002199817
Leotiomycetes	0.000195972	0.00040234	0.00098594	0	0	0.005303312	0.004970021	0	0
Pezizomycetes	0.000923559	0	0.00035067	0	0.003561019	0.000528413	0.002100875	0.002881958	7.04442E-05
Saccharomycetes	0.000576587	0	0.00031091	9.2539E-05	0	0.000779643	0.00013733	0.002000263	8.80553E-05
Sordariomycetes	0.004413693	0.00783718	0.00222537	0.008729	0	0.022805715	0.018874151	0.004362056	0.00453967
Agaricomycetes	0.005217147	0.02611444	0.01509592	0.00210531	0.002553168	0.005908241	0.019486455	0.012322624	0.006823802
Cystobasidiomycetes	0	0	0	0.00591164	0	0	0	0	0
Malasseziomycetes	6.6333E-05	0.00019423	8.0544E-05	3.365E-05	9.75622E-05	7.07204E-05	0.001522205	0	0
Tremellomycetes	0	0	0.00186515	0	0	0.001059387	0.000908191	0	3.21897E-05
Mortierellomycetes	0	0.00027002	0	0	0	0	0	0	0
Rozellomycotina	0	0	0	0	0	0.000967697	0	0	0
unclassified	0.729340428	0.69724161	0.44437153	0.67040543	0.93253809	0.891278354	0.855176875	0.726894225	0.502717466

Table S6. Unweighted-permanova data analysis was conducted for fungal bacterial communities in four periods. Specific evaluation parameters were shown in the table.

Healthy group-bacteria

Group 1	Group 2	Sample size	Permutations	pseudo-F	p-value	q-value
A	B	12	999	1.19379719	0.146	0.146
A	C	12	999	1.90947393	0.002	0.006
A	D	12	999	2.26252768	0.001	0.006
B	C	12	999	1.39293589	0.086	0.1032
B	D	12	999	1.77209408	0.004	0.0075
C	D	12	999	1.61386405	0.005	0.0075

Diseased group-bacteria

Group 1	Group 2	Sample size	Permutations	pseudo-F	p-value	q-value
A	B	12	999	1.53413578	0.035	0.035
A	C	12	999	2.23165862	0.018	0.0288
A	D	12	999	1.68385656	0.011	0.0288
B	C	12	999	2.2735224	0.023	0.0288
B	D	12	999	1.40173125	0.024	0.0288
C	D	12	999	2.39046314	0.008	0.0288

Healthy group-fungi

Group 1	Group 2	Sample size	Permutations	pseudo-F	p-value	q-value
A	B	12	999	1.05545888	0.326	0.489
A	C	12	999	0.77693211	0.906	0.906
A	D	12	999	2.37783951	0.005	0.012
B	C	12	999	1.00237994	0.421	0.5052
B	D	12	999	2.14025578	0.004	0.012
C	D	12	999	2.05297857	0.006	0.012

Diseased group-fungi

Group 1	Group 2	Sample size	Permutations	pseudo-F	p-value	q-value
A	B	12	999	0.87567365	0.75	0.75
A	C	12	999	1.56824199	0.002	0.006
A	D	12	999	2.06524003	0.009	0.0135
B	C	12	999	1.51759079	0.004	0.008
B	D	12	999	1.70784349	0.015	0.018
C	D	12	999	2.80369598	0.002	0.006

Figure S1 After being infected by *Gymnosporangium Yamadae*, apple leaves undergo a series of pathological changes both at the micro and macro level (Real shot pictures).

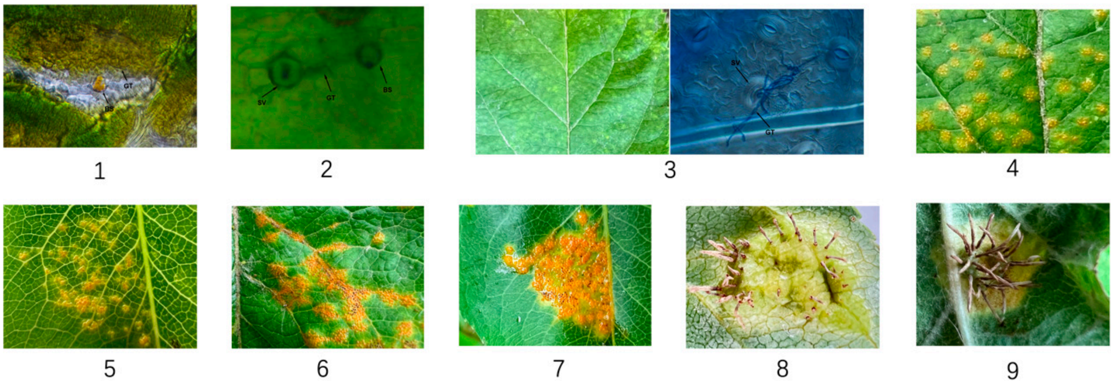

Figure S2 Rarefaction curves based on OTUs.

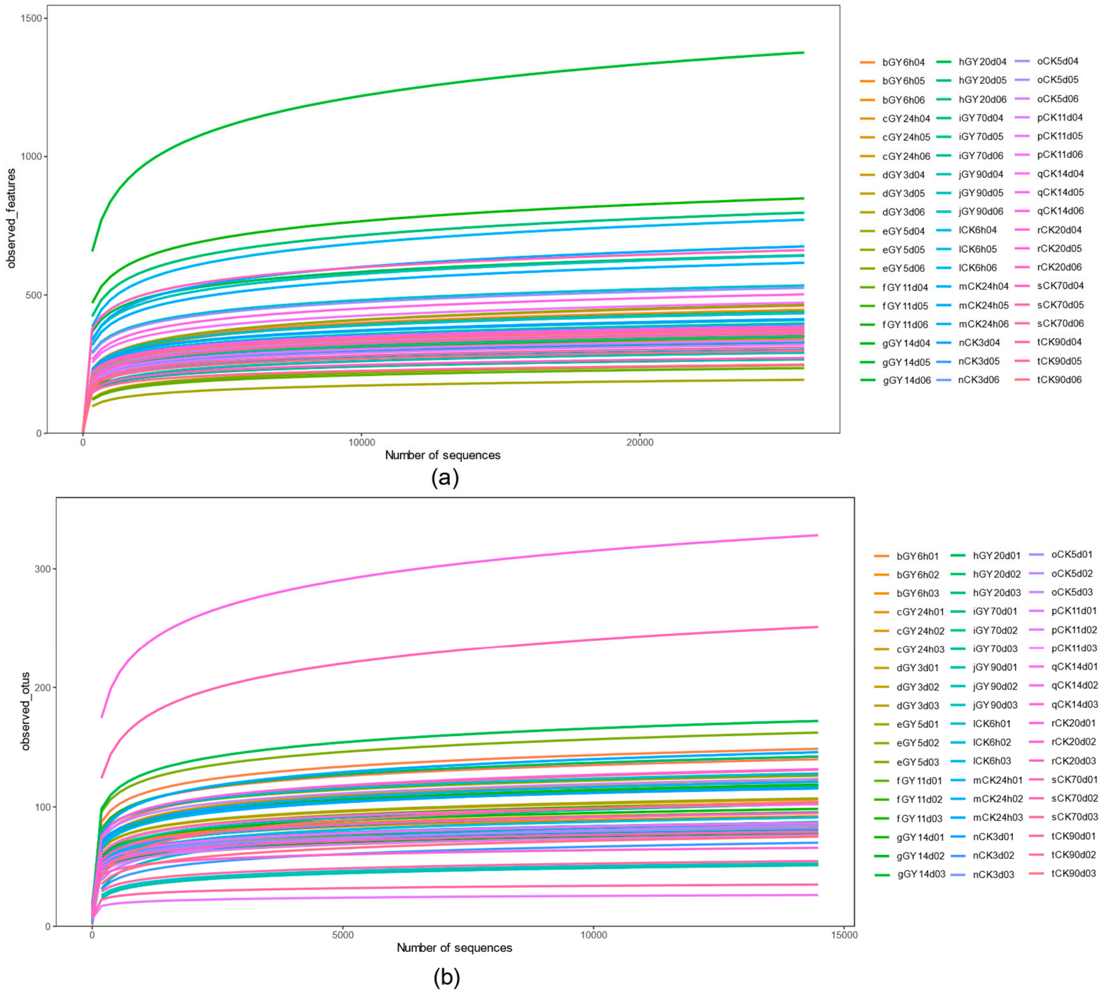

Figure S3 Histogram of sequence annotation degree of each sample at each

classification level. The X-axis (Sample Name) represents the sample name during sequencing, and the Y-axis (Sequence Number Percent) indicates the proportion of annotated sequences at each level compared to the total annotated data. The color order of the bars from top to bottom corresponds to the color order in the legend on the right. The maximum value at each taxonomic level is 1, representing 100% of the sequences that have been annotated at least at that level.

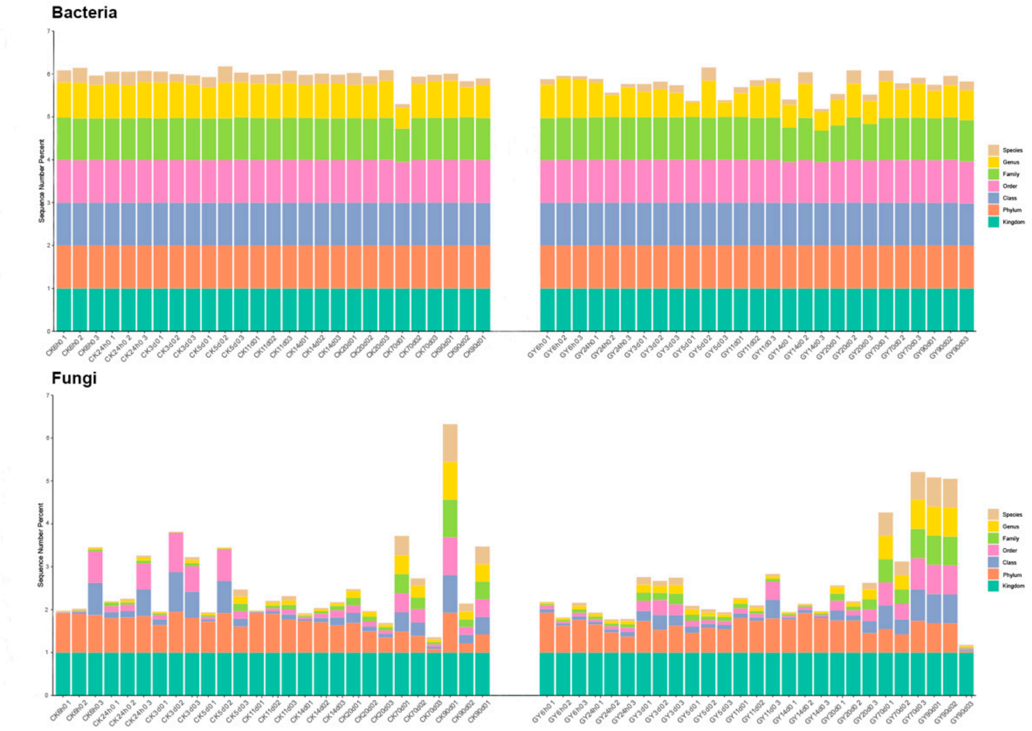

Figure S4 Histogram of the relative distribution of diseased and healthy samples at phyla and class levels.

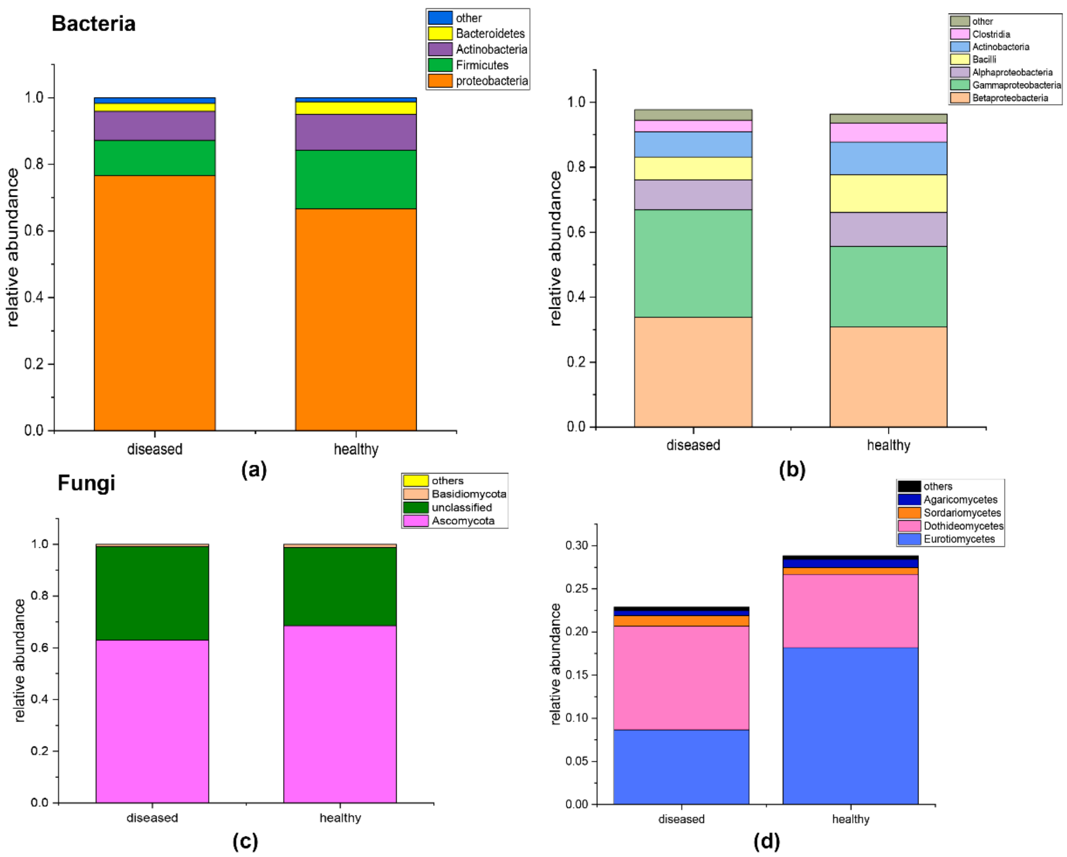

Figure S5 Changes in alpha diversity (indicated by Shannon index) of endophytic bacteria and fungi communities in healthy apple leaves and diseased leaves at 9 stages of infection were compared. Different letters represent statistically significant differences between stages, as determined by one-way Anova with *post hoc* Tukey's HSD test ($P<0.05$). The boxplots show the estimated range between the 25th and 75th percentiles, as well as the median, minimum, and maximum values of the observed data in each dataset.

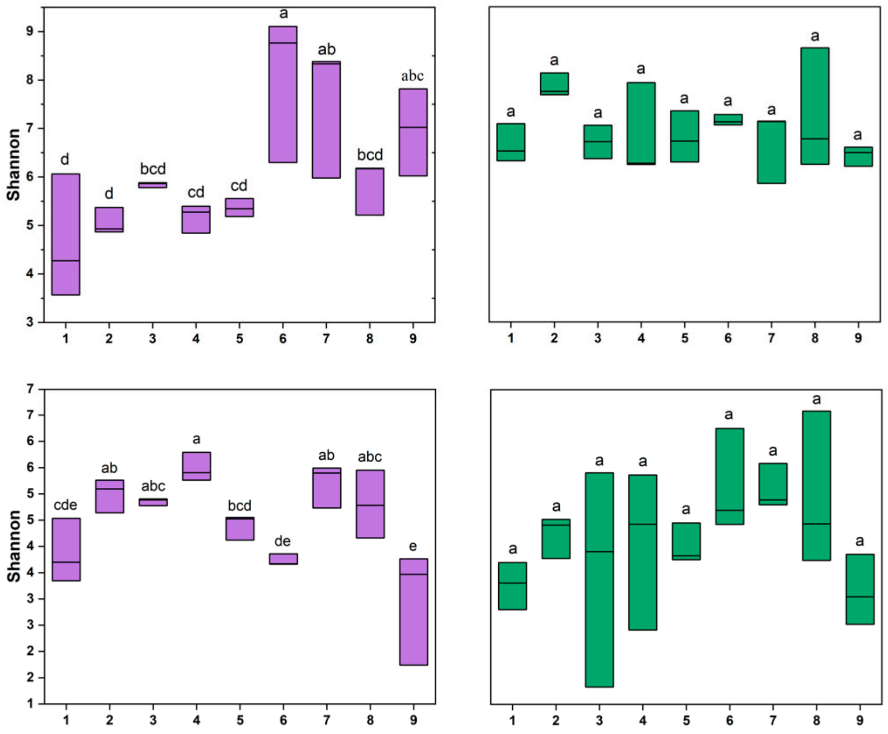

Figure S6 Bar chart of the number of OTUs at each stage for healthy samples and disease samples.

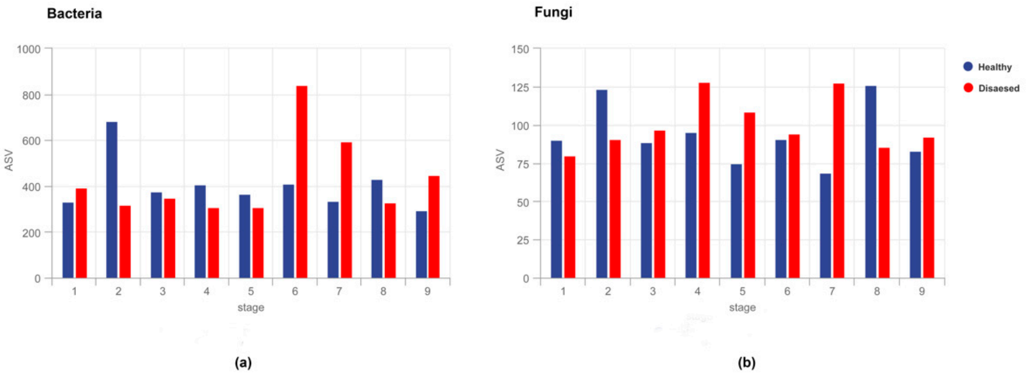

Figure S7 Taxonomic compositions of the leaves endophytic fungal community at the order level of the diseased and healthy groups.

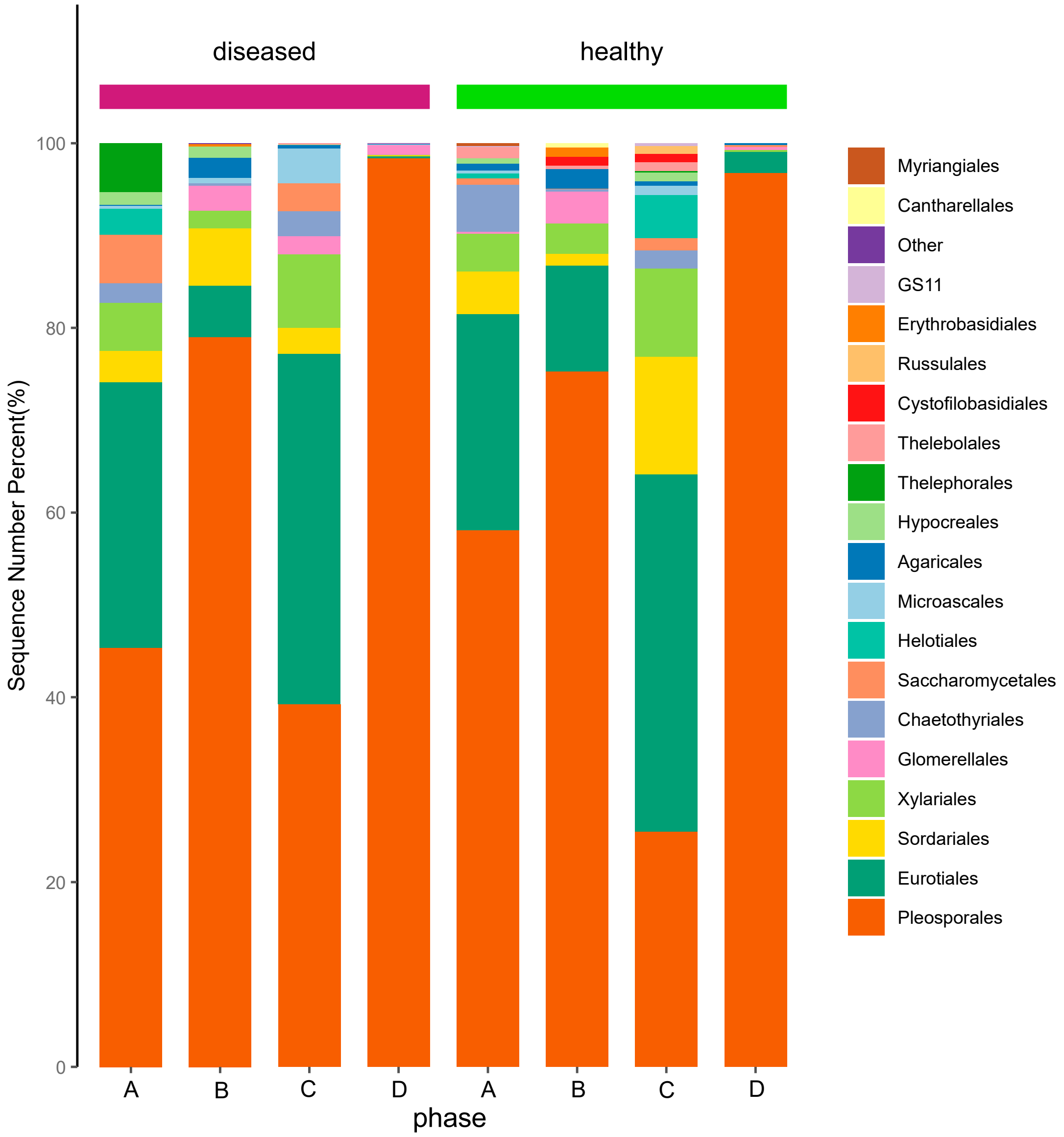

Figure S8 LEfSe Analysis LDA Barplot for endophytic bacteria. Each horizontal bar represents a species, and the length of the bar corresponds to the LDA value. A higher LDA value indicates a larger difference. The color of the bar corresponds to which group the species is a biomarker for, indicating higher abundance of the biomarker in the corresponding group.

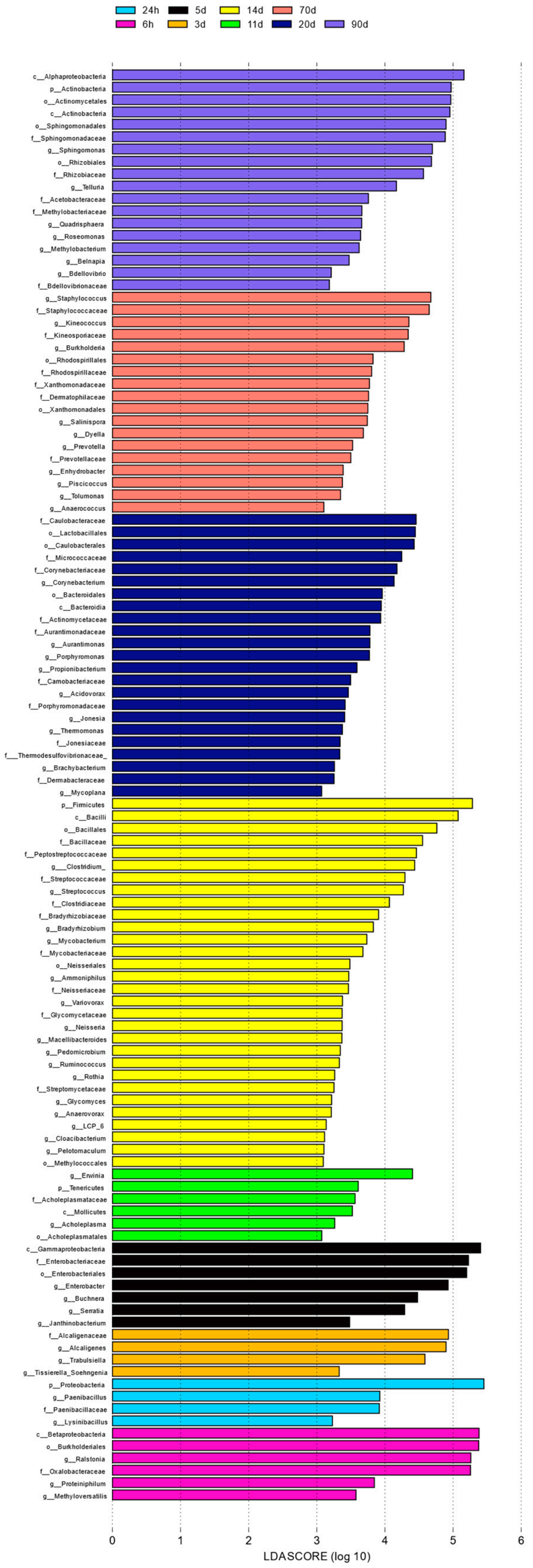

24h
6h
5d
3d
14d
11d
70d
20d
90d
c__Alphaproteobacteria
p__Actinobacteria
o__Actinomycetales
c__Actinobacteria
o__Sphingomonadales
f__Sphingomonadaceae
g__Sphingomonas
o__Rhizobiales
f__Rhizobiaceae
g__Telluria
f__Acetobacteraceae
f__Methylobacteriaceae
g__Quadrisphaera
g__Roseomonas
g__Methylobacterium
g__Belnapia
g__Bdellovibrio
f__Bdellovibrionaceae
g__Staphylococcus
f__Staphylococcaceae
g__Kineococcus
f__Kineosporiaceae
g__Burkholderia
o__Rhodospirillales
f__Rhodospirillaceae
f__Xanthomonadaceae
f__Dermatophilaceae
o__Xanthomonadales
g__Salinispora
g__Dyella
g__Prevotella
f__Prevotellaceae
g__Enhydrobacter
g__Piscicoccus
g__Tolumonas
g__Anaerococcus
f__Caulobacteraceae
o__Lactobacillales
o__Caulobacterales
f__Micrococcaceae
f__Corynebacteriaceae
g__Corynebacterium
o__Bacteroidales
c__Bacteroidia
f__Actinomycetaceae
f__Aurantimonadaceae
g__Aurantimonas
g__Porphyromonas
g__Propionibacterium
f__Carnobacteriaceae
g__Acidovorax
f__Porphyromonadaceae
g__Jonesia
g__Thermomonas
f__Jonesiaceae
f___Thermodesulfovibrionaceae_
g__Brachybacterium
f__Dermabacteraceae
g__Mycoplana
p__Firmicutes
c__Bacilli
o__Bacillales
f__Bacillaceae
f__Peptostreptococcaceae
g___Clostridium_
f__Streptococcaceae
g__Streptococcus
f__Clostridiaceae
f__Bradyrhizobiaceae
g__Bradyrhizobium
g__Mycobacterium
f__Mycobacteriaceae
o__Neisseriales
g__Ammoniphilus
f__Neisseriaceae
g__Variovorax
f__Glycomycetaceae
g__Neisseria
g__Macellibacteroides
g__Pedomicrobium
g__Ruminococcus
g__Rothia
f__Streptomycetaceae
g__Glycomyces
g__Anaerovorax
g__LCP_6
g__Cloacibacterium
g__Pelotomaculum
o__Methylococcales
g__Erwinia
p__Tenericutes
f__Acholeplasmataceae
c__Mollicutes
g__Acholeplasma
o__Acholeplasmatales
c__Gammaproteobacteria
f__Enterobacteriaceae
o__Enterobacteriales
g__Enterobacter
g__Buchnera
g__Serratia
g__Janthinobacterium
f__Alcaligenaceae
g__Alcaligenes
g__Trabulsiella
g__Tissierella_Soehngenia
p__Proteobacteria
g__Paenibacillus
f__Paenibacillaceae
g__Lysinibacillus
c__Betaproteobacteria
o__Burkholderiales
g__Ralstonia
f__Oxalobacteraceae
g__Proteiniphilum
g__Methyloversatilis
0
1
2
3
4
5
6
LDASCORE (log 10)

Figure S9 LEfSe Analysis LDA Barplot for endophytic fungi. Each horizontal bar represents a species, and the length of the bar corresponds to the LDA value. A higher LDA value indicates a larger difference. The color of the bar corresponds to which group the species is a biomarker for, indicating higher abundance of the biomarker in the corresponding group.

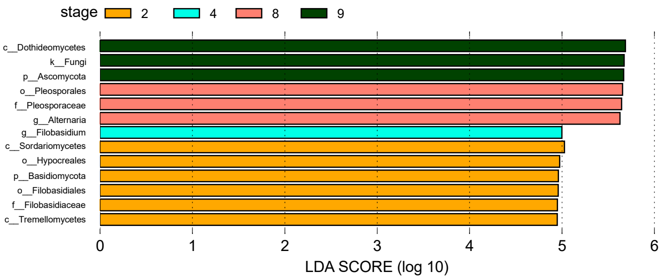

Figure S10 Relevant parameters of endophytic bacteria community co-occurrence network at different stages.

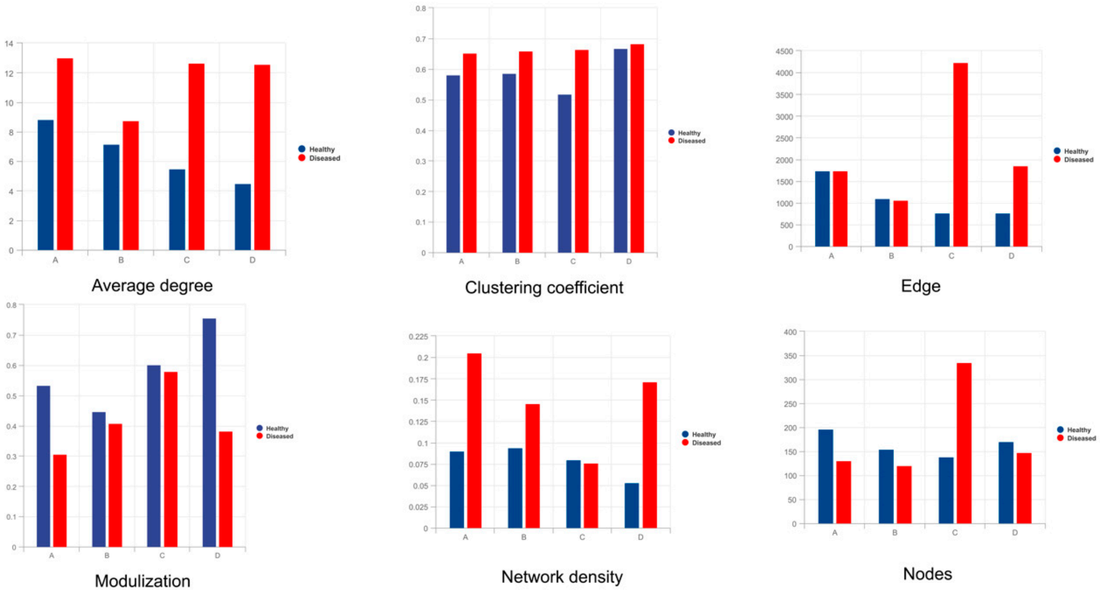

Figure S11 Relevant parameters of endophytic fungi community co-occurrence network at different stages.

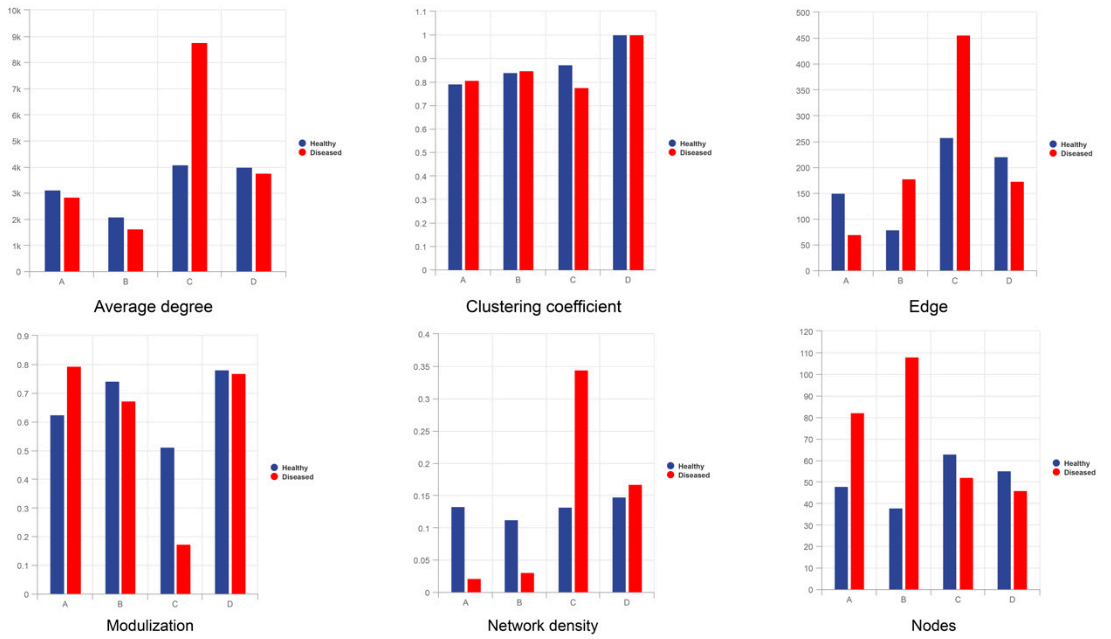

Figure S12 The functional enrichment of endophytic bacterial and fungal sequences on MetaCyc pathway in healthy and diseased apple leaves was demonstrated by PCA analysis.

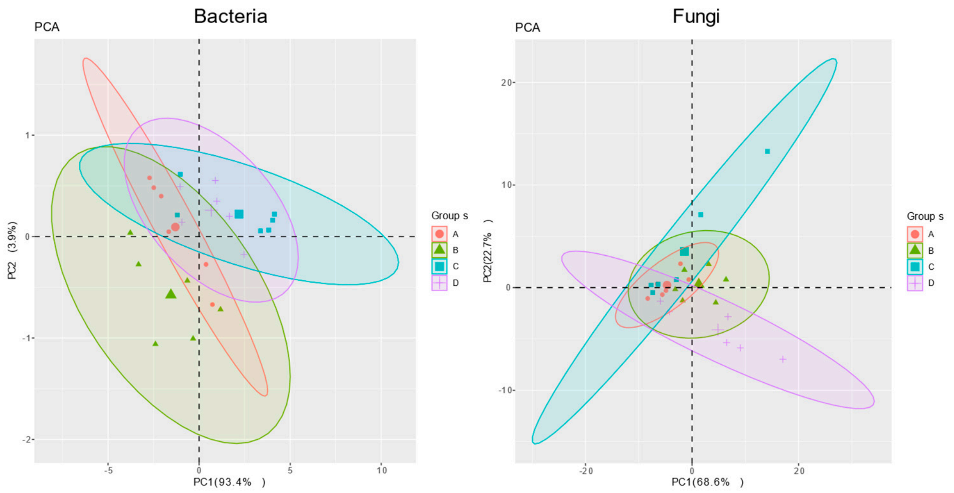

Figure S13 Functional categories in which fungal community sequences are significantly enriched at various phases. Different letters indicate statistically significant differences among stages.

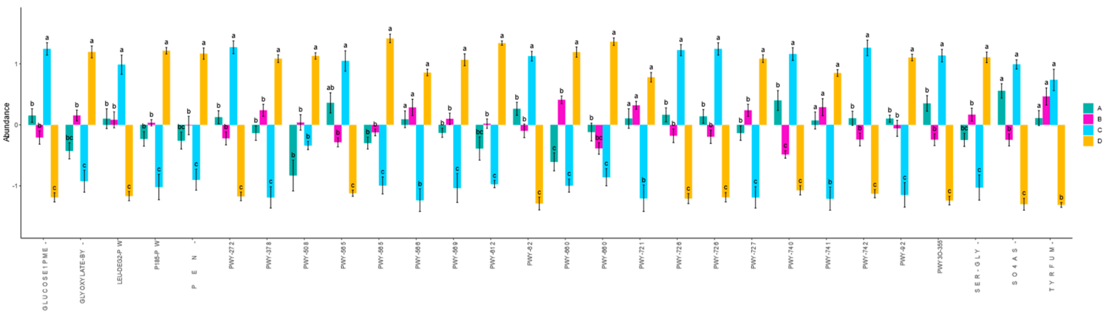
Abundance
1
0
-1
GLUCOSE1PME-
GLYOXYLATE-BY-
LEU-DEG2-PWY
P185-PWY
PEN-
PWY-272
PWY-378
PWY-508
PWY-565
PWY-565
PWY-566
PWY-569
PWY-612
PWY-62
PWY-660
PWY-660
PWY-721
PWY-726
PWY-726
PWY-727
PWY-740
PWY-741
PWY-742
PWY-92
PWY3O-355
SER-GLY-
SO4AS-
TYRFUM-
A
B
C
D
