# Supplementary material for: Time-Course Responses of Apple Leaf Endophytes to the Infection of Gymnosporangium yamadae
Source: J Fungi (Basel). 2024 Feb 3;10(2):128. doi: 10.3390/jof10020128 (PMC10890309; doi:10.3390/jof10020128)
Supplement: Supplementary file 1 [file jof-10-00128-s001.zip › Supplementary File S1.pdf]

For bacterial
Primer Design

Sequencing region	Primer names	Primer sequence
799F_1193R	799F	AACMGGATTAGATACCCKG
	1193R	ACGTCATCCCCACCTTCC

PCR Amplification Conditions
5×FastPfu Buffer .... 4 μl
2.5 mM dNTPs .... 2 μl
Forward Primer(5 μM) .... 0.8 μl
Reverse Primer(5 μM) .... 0.8 μl
FastPfu Polymerase .... 0.4 μl
BSA .... 0.2 μl
Template DNA .... 10 ng

Add ddH2O to .... 20 μl
PCR Instrument: ABI GeneAmp® 9700
PCR reaction parameters:
a. 1× (3 minutes at 95°C)
b. Circular number × (30 seconds at 95°C；30 seconds at Annealing temperature °C；45 seconds at 72°C)
c. 10 minutes at 72°C，10°C until halted by user
Two rounds of amplification

Primer 1: 799F-1392R, annealing temperature 55℃, 27 cycles.
Primer 2: 799F-1193R, annealing temperature 55℃, 13 cycles.

For fungi
Primer Design

Sequencing region	Primer names	Primer sequence
ITS1F_ITS2R	ITS1F	CTTGGTCATTTAGAGGAAGTAA
	ITS2R	GCTGCGTTCTTCATCGATGC

PCR Amplification Conditions

5×FastPfu Buffer .... 4 μl
2.5 mM dNTPs .... 2 μl
Forward Primer(5 μM) .... 0.8 μl
Reverse Primer(5 μM) .... 0.8 μl
FastPfu Polymerase .... 0.4 μl
BSA .... 0.2 μl
Template DNA .... 10 ng

Add ddH2O to .... 20 μl
PCR Instrument: ABI GeneAmp® 9700
PCR reaction parameters:
a. 1× (3 minutes at 95°C)
b. Circular number × (30 seconds at 95°C；30 seconds at Annealing temperature °C；45 seconds at 72°C)
c. 10 minutes at 72°C，10°C until halted by user
